# HPRT1 activity loss is associated with resistance to thiopurine in ALL

**DOI:** 10.18632/oncotarget.23405

**Published:** 2017-12-19

**Authors:** Fan Yang, Houshun Fang, Dan Wang, Yao Chen, Yonggong Zhai, Bin-Bing S. Zhou, Hui Li

**Affiliations:** ^1^ Beijing Key Laboratory of Gene Resource and Molecular Development, College of Life Sciences, Beijing Normal University, Beijing, China; ^2^ Key Laboratory of Pediatric Hematology and Oncology Ministry of Health, Department of Hematology and Oncology, Shanghai Children's Medical Center, Shanghai Jiao Tong University School of Medicine, Shanghai, China; ^3^ Pediatric Translational Medicine Institute, Shanghai Jiao Tong University School of Medicine, Shanghai, China; ^4^ Department of Pharmacology and Chemical Biology, School of Basic Medicine and Collaborative Innovation Center for Translational Medicine, Shanghai Jiao Tong University School of Medicine, Shanghai, China

**Keywords:** thiopurine, leukemia, drug resistance, HPRT1 mutation, purine metabolism

## Abstract

Acute lymphoblastic leukemia (ALL) is an aggressive hematological tumor resulting from the malignant transformation of lymphoid progenitors. Thiopurine is a widely used drug in the maintaining treatment of ALL. After a period of chemotherapy, 20% of pediatric patients and over 50% of adult patients will relapse. To investigate the mechanisms of drug resistance *in vitro*, we established the thiopurine resistant cell lines Reh-6MPR (6-MP Resistant cell) and Reh-6TGR (6-TG Resistant cell) by stepwise selection of the ALL cell line Reh. Cell viability assay revealed that 6MPR and 6TGR cells were almost 1000-fold more resistant to thiopurine comparing with the control Reh cells, and thiopurine conversion was significantly impaired in the resistant cells. Mechanistically, a same novel hypoxanthine phosphoribosyl transferase 1 (HPRT1) mutation c.495_496insA (p.V165fs) was found by whole exome sequencing in both resistant cells. The HPRT1 mutation dramaticly decreased the production of [^13^C_5_,^15^N_4_]-IMP from [^13^C_5_,^15^N_4_]-hypoxanthine (HX), showed a loss-of-funciton mechanism. Notably, re-expression the wildtype HPRT1 in Reh-6MPR cell can reverse the drug resistance and thiopurine conversion in Reh-6MPR cells. These results highlight the importance of HPRT1's activity in thiopurine resistance.

## INTRODUCTION

Acute lymphoblastic leukemia (ALL) is the most common childhood cancer. The survival rates of childhood ALL have been improved with the development of risk-stratified combination chemotherapy. Though patients with relapsed ALL generally receive more intense treatment, relapsed ALL is still the leading cause of childhood cancer death, with cure rates of less than 40%, owing to intrinsic drug resistance [1–4]

The thiopurines 6-mercaptopurine (6-MP) and 6-thioguanine (6-TG) are key drugs in the treatment of ALL, administered to induce remission and used during maintenance therapy [5]. Both of them are prodrugs, which require intracellular conversion into active compounds by purine salvage pathway enzyme hypoxanthine phosphoribosyl transferase 1 (HPRT1). 6-MP and 6-TG are converted by HPRT1 into thioinosine monophosphate (TIMP) and thioguanosine monophosphate (TGMP), respectively, and subsequently into the active cytotoxic thioguanine nucleotides (TGNs), which are incorporated into DNA during DNA replication and exhaust the DNA repair machinery by inducing repeated mismatch repair. Therefore the activity of HPRT1 can affect the therapeutic efficacy of these drugs [6]. However, which kind of HPRT1 mutation will cause drug resistance is not fully understand.

To investigate the mechanisms of drug resistance in ALL, we established the resistant cell lines Reh-6MPR (6-MP Resistant cell) and Reh-6TGR (6-TG Resistant cell) by inducing Reh cells with 6-MP or 6-TG, and we found a novel HPRT1 mutation c.495_496insA (p.V165fs) in these two resistant cells by whole exome sequencing. In control cells, HPRT1 convert the thiopurines to TIMP and TGMP. But in the resistant cell lines, the function of HPRT1 is impaired and the metabolism of thiopurines is defected. The 6MPR and 6TGR cells shows 1000-fold more resistant to thiopurine comparing with control Reh cells. Re-expression of wildtype HPRT1 in these cells could reverse the drug resistance phenotype. Our results indicate a new resistance mechanism and potential therapeutic strategies to overcome thiopurine resistance.

## RESULTS

### Establishment of the thiopurine resistance cell lines Reh-6MPR and Reh-6TGR

**Figure 1:**
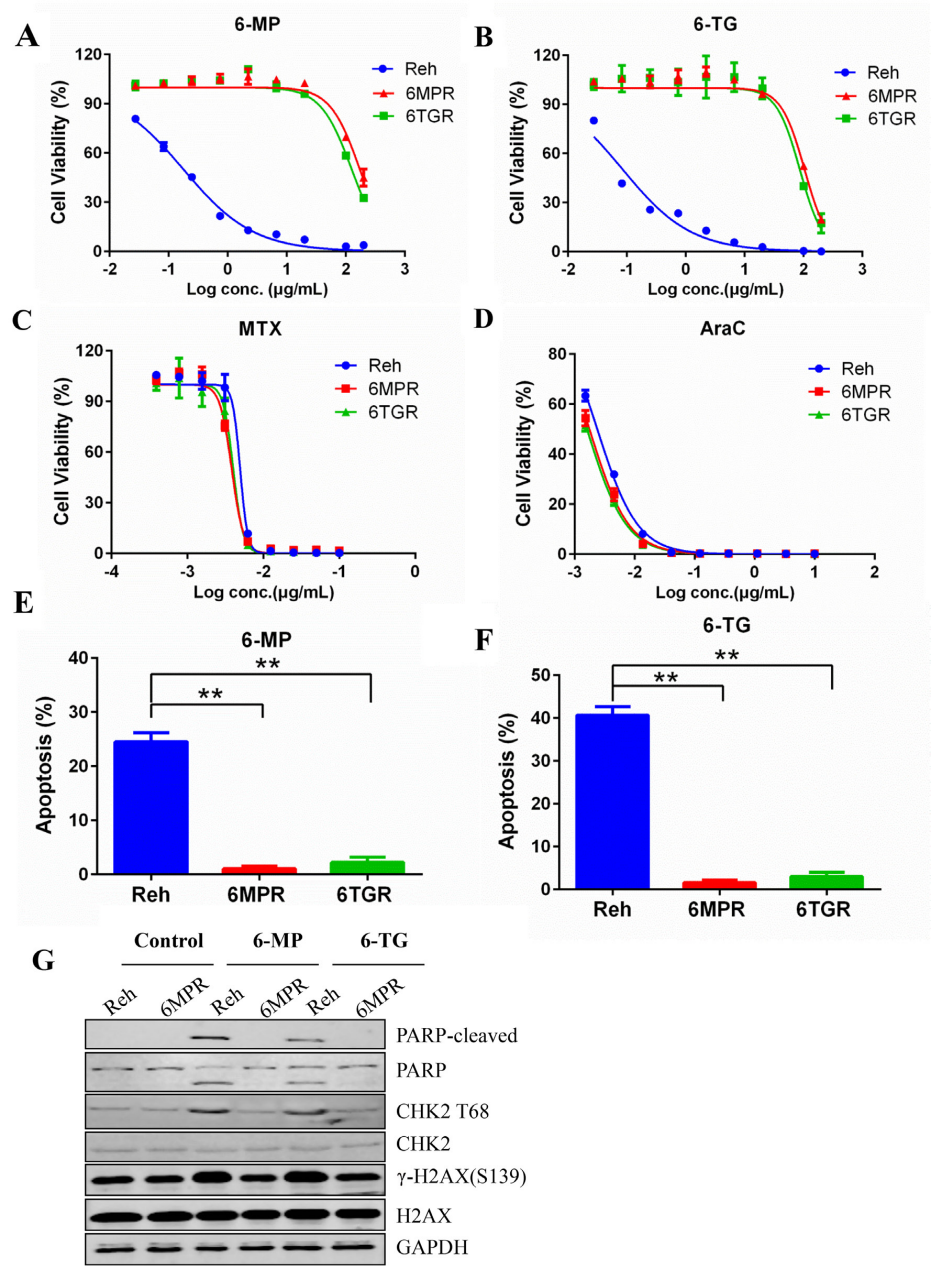
Establishment of the thiopurine resistant cell lines Reh-6MPR and Reh-6TGR **(A, B, C** and **D)** Drug sensitivity of 6-MP, 6-TG, MTX and AraC were analyzed with Cell TiterGlo assay. **(E, F)** Apoptosis induced by 6-MP and 6-TG was analyzed with annexin V-PE apoptosis detection Kit. Data are expressed as mean ± SD. ^**^P<0.01 compared with ctrl Reh group. **(G)** Western blot of DDR markers (PARP-cleaved, P-CHK2 and γ-H2AX).

To investigate the mechanisms of cellular resistance to thiopurine in ALL, we induced thiopurine-resistant cell lines from the Reh human ALL cells. Reh cells were selected by stepwise increasing concentrations of 6-MP or 6-TG during a 3-months period. Finally, we established two resistant cell lines Reh-6MPR and Reh-6TGR. Reh-6MPR and Reh-6TGR cells showed stable resistant phenotypes at least for 6 months after continued growth without drugs. Cell viability assay indicated that Reh-6MPR cells were 968- and 1161-fold more resistant to 6-MP and 6-TG respectively, compared with control Reh cells culturing for the same long time. Similarly, the Reh-6TGR cells were 699- and 956- fold more resistant to 6-MP and 6-TG than control Reh respectively (Table 1, Figure 1A and 1B). Interestingly, the Reh-6MPR and Reh-6TGR cells were still as sensitive as control Reh cells to other chemotherapeutic drugs that are used in ALL treatment, such as methotrexate (MTX) and cytosine arabinoside (AraC) (Table 1, Figure 1C and 1D). That leads to the suggestion that the resistance of these two cell lines is specific to 6-MP and 6-TG.

**Table 1:** IC50 of anticancer drugs in Reh cells and resistant cells

Drugs	IC50 (μg/mL)			^a^Relative resistance	^b^Relative resistance
	Reh	Reh-6MPR	Reh-6TGR		
6-MP	0.18±0.02	174.23±16.75^***^	125.73±3.54^***^	968	699
6-TG	0.09±0.01	104.50±2.79^***^	86.05±5.82^***^	1161	956
MTX	0.005±0.0006	0.0039±0.0001	0.004±0.0002	0.78	0.80
AraC	0.0024±0.0001	0.0018±0.0002	0.0016±0.0001	0.75	0.67

Values are given as mean ± SD. IC50 of resistant cells to 6-MP and 6-TG differed significantly from Reh cells, ^***^P<0.001 compared with Reh group. ^a^Relative resistance was obtained by dividing the IC50 mean value of Reh cells by the IC50 value of Reh-6MPR cells; ^b^Relative resistance was obtained by dividing the IC50 mean value of Reh cells by the IC50 value of Reh-6TGR cells.

Thiopurines exert their cytotoxicity primary by incorporating TGNs into DNA, leading to DNA damage response (DDR) and apoptosis. We found that much less Reh-6MPR and Reh-6TGR cells went apoptosis when inducing by thiopurines than the Reh cells (Figure 1E and 1F). Moreover, the expression of the DDR biomarkers γ-H2AX, the phospho-CHK2 (T68) and the apoptosis biomarker cleaved PARP were much lower in the resistant cell line Reh-6MPR than those in control Reh cells, consistent with reduced DNA damage (Figure 1G).

### The thiopurine metabolism was impaired in the resistant cells

**Figure 2:**
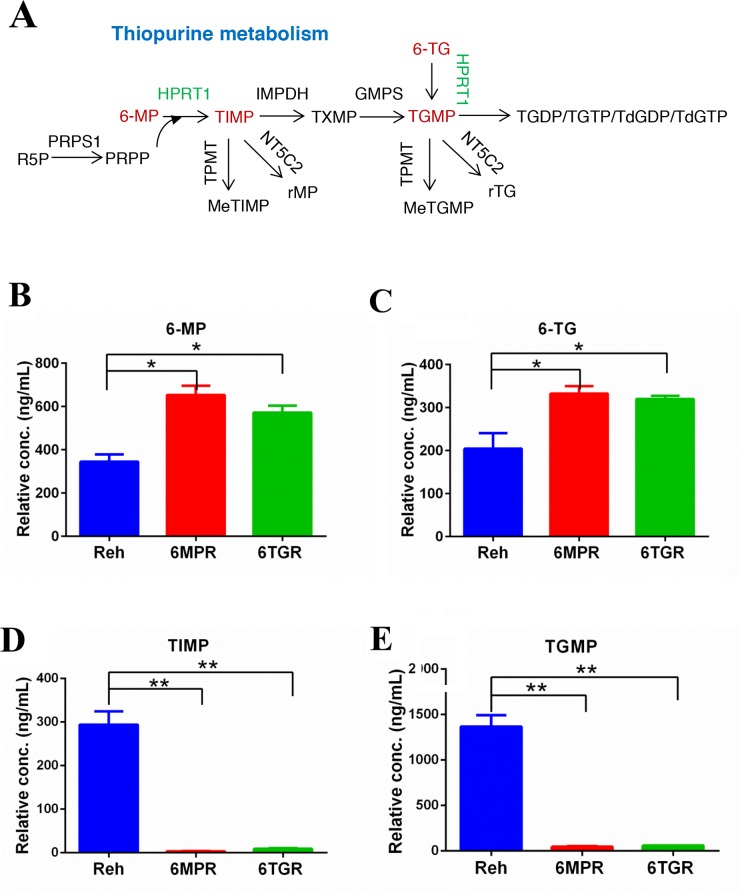
Thiopurine metabolism in Reh cells and resistant cells **(A)** The diagram of thiopurine metabolism. **(B, C)** The accumulations of 6-MP and 6-TG were measured by LC-MS in ctrl Reh cells and resistant cells. Data are expressed as mean ± SD. ^*^P<0.05 compared with ctrl Reh group. **(D, E)** Drug metabolites (TIMP and TGMP) were measured by LC-MS in ctrl Reh cells and resistant cells. Data are expressed as mean ± SD. ^**^P<0.01 compared with ctrl Reh group.

To determine how the resistant cells avoid thiopurine-induced DDR, we analyzed the thiopurine metabolism in resistant cells and control Reh cells. 6-MP and 6-TG are converted by HPRT1 into TIMP and TGMP, respectively, subsequently converted by the other purine salvage pathway enzymes into the active TGNs, which incorporated into DNA (Figure 2A). We used LC-MS to measure the levels of 6-MP, 6-TG, TIMP and TGMP, which representing thiopurine metabolism in cells. The results revealed that the 6-MP or 6-TG were accumulated in the resistant cells (Figure 2B and 2C), and the thiopurine metabolites TIMP and TGMP were markedly reduced in resistant cells compared with Reh cells (Figure 2D and 2E). These results suggested that the resistant cells can not convert thiopurine into active TGNs and exert resistance to drug-induced DDR and apoptosis.

### HPRT1 loss of function mutation confers to the resistance to thiopurines

**Figure 3:**
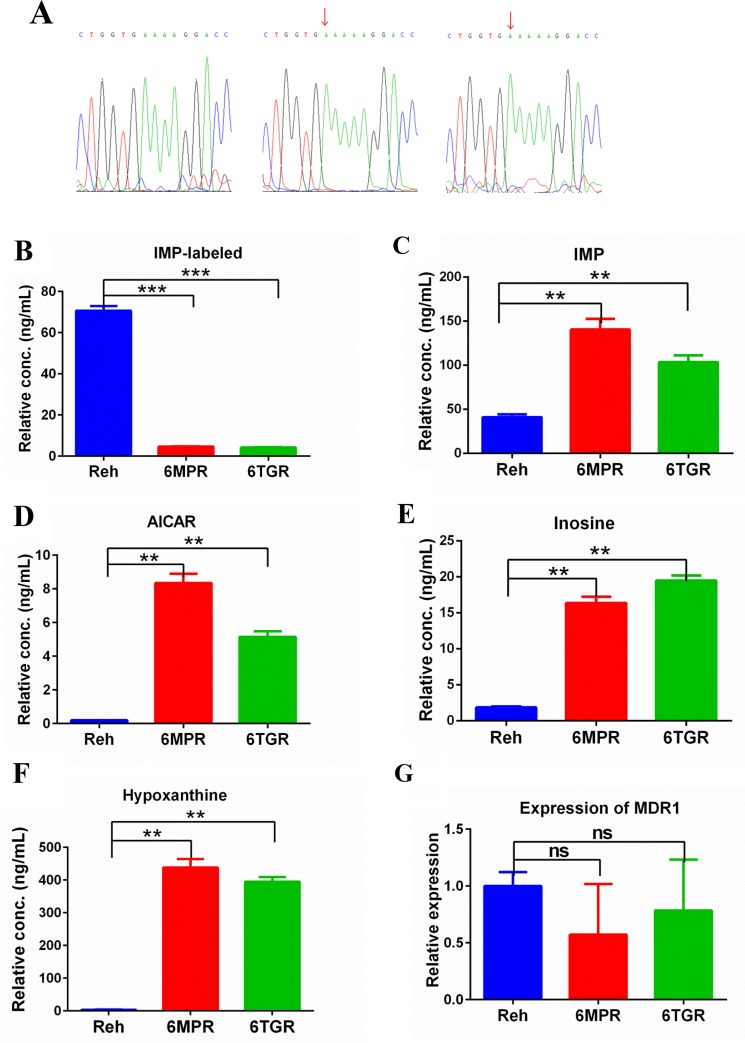
Purine metabolism in Reh cells and resistant cells **(A)** Identification HPRT1 mutation in resistant cells by Sanger sequencing. **(B)** After incubation with [^13^C_5_,^15^N_4_]-hypoxanthine for 4h, labeled-IMP was measured by LC-MS in Reh cells and resistant cells. Data are expressed as mean ± SD. ^***^P<0.001 compared with ctrl Reh group. **(C, D, E, F)** The metabolites (IMP, AICAR, Inosine and HX) were measured by LC-MS in Reh cells and resistant cells. Data are expressed as mean ± SD. ^**^P<0.01 compared with ctrl Reh group. **(G)** The expression of MDR1 was detected by using Real-time quantitative RT-PCR assay. Data are expressed as mean ± SD.

To explore the mechnism of the drug resistance in Reh-6MPR and Reh-6TGR cells, we performed whole exome sequencing in the resistant and control Reh cells (Supplementary Tables 1 and 2). Interestingly, we found a novel HPRT1 mutation at the same site [c.495_496insA (p.V165fs)] in Reh-6MPR and Reh-6TGR cells. The mutation causes the HPRT1 frameshift and premature termination. To confirm the results, we performed sanger sequencing in the resistant and control Reh cells. As shown in Figure 3A, HPRT1 was mutated in Reh-6MPR and Reh-6TGR cells but wildtype in control Reh cells. To our knowledge, this mutation has not been reported before [7, 8]. Furthermore there were no thiopurine S-methyltrasferase gene (TPMT) [9], phosphoribosyl pyrophosphate synthetase 1 gene (PRPS1) [10] and cytosolic 5’-nucleotidase II gene (NT5C2) [11] mutations in Reh-6MPR and Reh-6TGR cells (Supplementary Tables 1 and 2), which previously shown to be associated with thiopurine resistance in ALL.

To test the functional effect of HPRT1 mutaion, we measured the HPRT1 activity in the Reh-6MPR, Reh-6TGR and Reh cells by following the generation of [^13^C_5_,^15^N_4_]-IMP from [^13^C_5_,^15^N_4_]-hypoxanthine (HX). The results showed that the HPRT1 activity was significantly decreased in the resistant cells, consistent with the HPRT1 mutation is a loss of function mutation (Figure 3B). According to the protein structure study, there were 11 active sites in the conserved Phosphoribosyl transferase (PRT)-type I domain [12]. The frameshift from V165 could only influence the last active site. Our data indicated that the last active site is essential for the function of PRTases type I domain or there is still another domain, which is essential for the function of HRPT1. Furthermore, we measured the *de novo* purine biosynthesis pathway's activity. To our surprise, the nucleotides IMP, 5-amimoimidazole-4- carboxamide-1-β-d-ribofuranoside (AICAR), Inosine and HX were dramatically increased in the resistant cell lines, suggested that the *de novo* purine synthesis pathway was hyper activated in the resistant cells (Figure 3C-3F).

Meanwhile, we tested the expression of multidrug resistance gene 1 (MDR1) [13, 14] which is a direct active transporter for a variety of drugs. There was no significant difference between Reh cells and the resistant cells, consistent with 6-MP or 6-TG accumulation in resistant cells (Figure 3G). Reading frame shift and premature termination usually cause mRNA premature degradation, so we also measured the mRNA and protein levels of HPRT1 in the resistance cells. As shown in Supplementary Figure 1A and 1B, the mRNA and protein level of HPRT1 were both significantly reduced in the Reh-6MPR and Reh-6TGR cells compared to control Reh cells. These results suggested that the thiopurine uptake is normal but the thiopurine conversion is impaired by HPRT1 loss of function mutation in the resistant cells.

### Re-expression of wildtype HPRT1 can reverse the thiopurine resistant phenotype in Reh-6MPR cells

**Figure 4:**
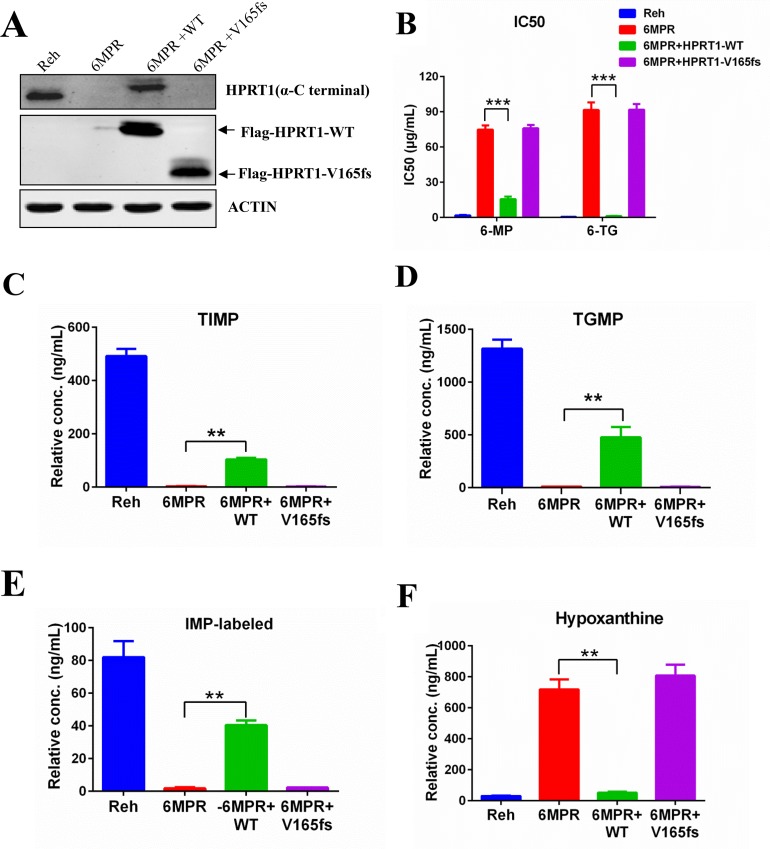
HPRT1-wt can reverse the resistance in Reh-6MPR cells **(A)** The expression levels of HRPT1 were detected by western blot. **(B)** 6-MP and 6-TG IC50 values of Reh-6MPR cells and HPRT1 re-expression cells. Data are expressed as mean ± SD. ^***^P<0.001 compared with ctrl Reh group. **(C, D, E, F)** The TIMP, TGMP, labeled-IMP and hypoxanthine were measured in Reh-6MPR cells and HPRT1 re-expression cells by LC-MS. Data are expressed as mean ± SD. ^**^P<0.01 compared with ctrl Reh group.

To test whether loss the activity of HPRT1 is specifically contribute to the drug resistance phenotype, we transfected wildtype or mutant HPRT1 expression cassete to the resistance cell line Reh-6MPR (Figure 4A). Expression of wildtype HPRT1 could reverse the thiopurine resistant phenotype of resistant cells (Figure 4B). Wildtype HPRT1 overexpression also restored the thiopurine conversion in Reh-6MPR cells (Figure 4C, 4D and Supplementary Figure 2), which is consistent with the activity of HPRT1 in those cells (Figure 4E). We also found that expression of wildtype HPRT1 could suppress the *de novo* purine synthesis pathway up-regulation in the resistant cells (Figure 4F and Supplementary Figure 3A). On the contrary, re-expression of HPRT1 V165fs mutant shows little effect in thiopurine sensitivity, drug metabolism and purine synthesis regulation (Figure 4B-4F and Supplementary Figure 3A). Taking together, our results lead to the assumption that the activity of HPRT1 plays an important role in thiopurine resistance.

### Knockdown HPRT1 induce thiopurine resistance

**Figure 5:**
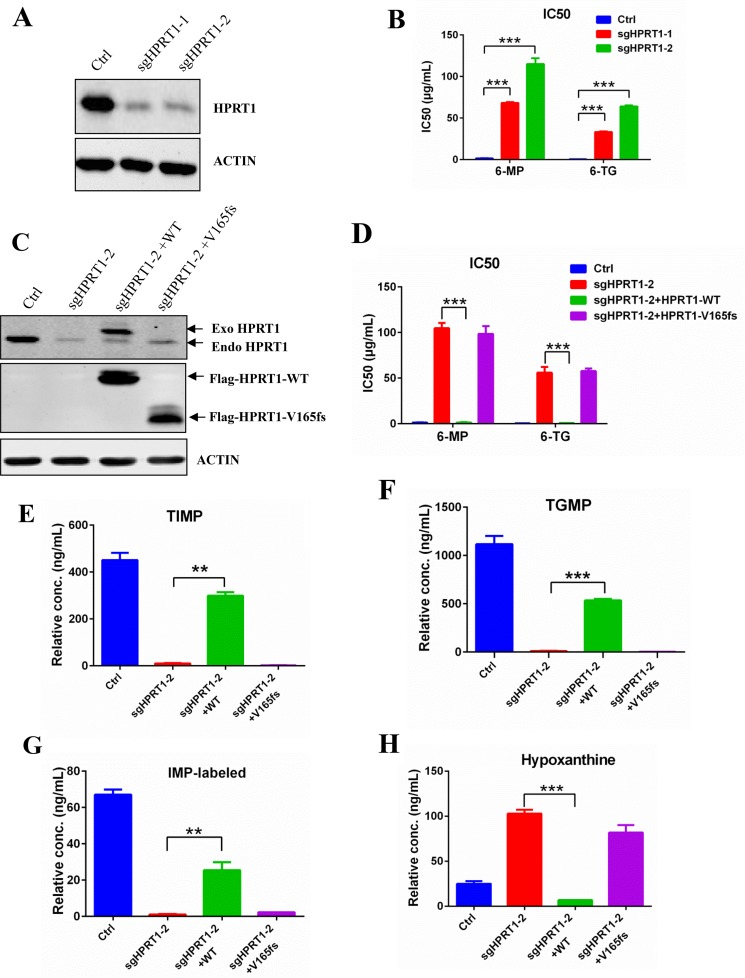
Knockdown HPRT1 induce thiopurine resistance **(A)** Western blot of HPRT1 in the knockdown cells. **(B)** 6-MP and 6-TG IC50 values of HPRT1 knockdown cells. Data are expressed as mean ± SD. ^***^P<0.001 compared with ctrl Reh group. **(C)** The expression levels of endogenous and exogenous HRPT1 were detected by western blot. **(D)** 6-MP and 6-TG IC50 values of knockdown cells with HPRT1 re-expression. Data are expressed as mean ± SD. ^***^P<0.001 compared with ctrl Reh group. **(E, F, G, H)** The TIMP, TGMP, labeled-IMP and hypoxanthine were measured in HPRT1 knockdown cells and HPRT1 re-expression cells by LC-MS. Data are expressed as mean ± SD. ^***^P<0.001, ^**^P<0.05 compared with ctrl Reh group.

To further confirm the role of HPRT1 in thiopurine drug resistance, we used CRISPR-Cas9 technology to knockdown endogenous HPRT1 in Reh cells (Figure 5A). The IC50 of 6-MP and 6-TG were dramatically increased in HPRT1 knockdown cells compared to control Reh cells (Figure 5B). Re-expression of wildtype HPRT1 (sgRNA resistent) in the knockdown cells can effectively reversed not only the thiopurine resistant phenotype (Figure 5C and 5D), but also the thiopurine metabolism (Figure 5E, 5F and Supplementary Figure 4). However, re-expression of mutated HPRT1-V165fs (sgRNA resistent) did not show the same effect in thiopurine resistant and thiopurine metabolism (Figure 5C-5F and Supplementary Figure 4). We also used IMP-labeled level to indicate the HPRT1 activity in the re-expression cells and HPRT1-V165fs show no enzyme activity (Figure 5G). These results suggest that HPRT1-V165fs is a loss-of-function mutant. Consistent with the observation in resistant cells, *de novo* purine synthesis pathway was also up regulated when knocking down endogenous HPRT1 (Figure 5H and Supplementary Figure 3B). Expression of wildtype HPRT1 suppressed the increasing of hypoxanthine, but expression of HPRT1-V165fs did not (Figure 5H).

## DISCUSSION

Many mechanisms of modulating thiopurine conversion have been reported: methylation by TPMT, clearance of cytotoxic nucleotides by relapse-specific NT5C2 mutations, competitive inhibition of thiopurine activation by feedback-defective PRPS1 mutations and so on [9–11]. In our study, we did not find TPMT, NT5C2 and PRPS1 mutations in the resistant cell lines, but we identified a novel HPRT1 mutation V165fs. Although many HPRT1 mutations have been found, HPRT1 mutation V165fs was not reported previously. And we identified its loss-of-function mutation and the important role of HPRT1 in thiopurine resistance in ALL through functional analysis.

In thiopurine metabolism, HPRT1 catalyzes the conversion of 6-MP and 6-TG to its active TGNs metabolites [5]. 6-MP and 6-TG were accumulated and its metabolites TIMP and TGMP were markedly decreased in Reh-6MPR cells. Also, the expression of MDR1 in resistant cells and Reh cells was no significantly different between the two cell lines. These results suggested that the thiopurine uptake is normal but the thiopurine conversion is impaired in the resistant cells.

HPRT1 is a purine salvage enzyme that catalyzes the conversion of hypoxanthine and phosphoribosyl pyrophosphate (PRPP) to IMP [6]. In thiopurine resistant cells, HPRT1 mutation V165fs failed to convert [^13^C_5_,^15^N_4_]-hypoxanthine to [^13^C_5_,^15^N_4_]-IMP, and when we expressed HPRT1 V165fs mutant in wildtype Reh cells, it could not block the wildtype HPRT1 function (data not shown). These results indicated that the HPRT1-V165fs is a loss-of-function mutation, which excludes the domain negative mechanism.

We observed that the purine biosynthesis intermediates IMP, HX, AICAR and Inosine were increased in the resistant cells, which suggests *de novo* purine synthesis pathway is up-regulated. The loss-of-function mutant HPRT1-V165fs fails to convert thiopurine, meanwhile it impairs the activity of purine salvage pathway in resistant cells. Thus the resistant cells need to up-regulate the *de novo* purine biosynthesis pathway to supply sufficient nucleotide pools to maintain DNA replication and cell proliferation [15, 16].

In conclusion, the present results of this study show that we found the novel HPRT1 mutation V165fs causing loss-of-function of HPRT1 and that the mutation plays a critical role in thiopurine resistance in ALL.

## MATERIALS AND METHODS

### Cell culture

The parental cell line Reh and resistant cell lines Reh-6MPR and Reh-6TGR were cultured in RPMI 1640 medium supplemented with 10% FBS, 100 U/ml penicillin G and 100 μg/ml streptomycin. Reh-6MPR and Reh-6TGR cells were selected from Reh cells by treating with stepwised increasing concentrations of 6-MP or 6-TG during a 3-month period. All cells were incubated at 37°C in 5% CO2.

### Cell viability

Drug sensitivity assay was performed as described previously [10]. Briefly, cells were seeded in 96-well plates (12,000 cells per well) in 0.1 ml medium and treated for 72 h with serially diluted anticancer drugs. CellTiter-Glo (CellTiter-Glo Luminescent kit, Promega) reagents (50 μl) was added to each well and mixed for 10 min before the luminescence was measured on a microplate reader (Biotek, USA).

### Apoptosis assay

Cells were seeded in triplicate in 24-well plates (5×10^5^ cells per well) and treated for 72h with 6-MP (10 μg/ml) or 6-TG (10 μg/ml). Apoptosis was measured by staining with Annexin V–PE and 7-AAD (AnnexinV-PE Apoptosis Detection kit, BD Pharmingen, cat. No.:559763) followed by flow cytometry on a FACS flow cytometer (BD, Canto II).

### Analyze of accumulation and metabolites of 6-MP and 6-TG

Cells were cultured at a density of 5×10^5^ cells per ml for 4 h in RPMI 1640 containing 10 μM 6-MP or 10 μM 6-TG, then harvested the cells and assayed by a modified method based on what was previously described [10]. Intracellular accumulation of 6-MP and 6-TG and its metabolites (TIMP and TGMP) were determined by LC-MS. The relative concentration was defined according to the standard curve of compound dissolved in 80% methanol.

### Purine metabolism in Reh cells and resistant cells

Purine metabolism assay was performed as described previously [10]. Briefly, cells were cultured in RPMI 1640 at a density of 1 × 10^6^ cells per ml, after addition [^13^C_5_,^15^N_4_]-hypoxanthine (Cambridge Isotope Laboratories, cat. No. CNLM-7894-PK), cells were cultured for an additional 4 h, then harvested and pelleted. The reaction was quenched in cold 80% methanol, cells were centrifuged at 15,000 r.p.m. for 15 min and metabolites (IMP, AICAR, Inosine and HX) in the supernatant were analyzed by LC-MS [17]. IMP synthesis through the purine salvage pathway was measured by ^13^C_5_,^15^N_4_ incorporation (molecular weight peak IMP-labeled); The relative concentrations were defined according to the standard curve of compounds dissolved in 80% methanol without correcting for cell matrix effect.

### Whole-exome sequencing and analysis

Whole-exome capture libraries were prepared according to standard protocols using SureSelect Human All Exon 50Mb and 38Mb kit (Agilent technologies). We performed paired-end (2 × 100 bp) sequencing using the Illumina HiSeq2000 instruments, imaging analysis and base calling using the Illumina Real Time Analysis (RTA) Pipeline version 1.9. After removing reads with sequence matching the sequencing adaptors and low-quality reads (reads with more than 50% of bases with Phred quality score of < 5), high-quality reads were aligned to the reference human genome (hg19, http://genome.ucsc.edu/) using Burrows-Wheeler analysis (BWA) with default parameters. The reference human genome assembly hg19 was used for mapping all samples. SNVs were called with GATK and filtered with recommended threshold (SNV quality ≥ 20, > 3 reads covered and depth ≥ 10).

### Lentivirus infection and western blot analysis

The human HPRT1 coding region was cloned into pGV320 mcherry vector (Shanghai GeneChem) and confirmed by DNA sequencing. The lentiviral constructs were packaged as described previously [10]. The virus was transfected into cells supplemented with 8 μg/ml Polybrene (Sigma). The medium was changed 24 h after infection, and cherry-positive cells were sorted with a Moflo XDP (Beckman Counter). Cells were harvested in lysis buffer and analyzed by SDS–PAGE with the following antibodies: anti-flag (cat. No.M8823, 1:5,000 dilution, Sigma, USA), β-actin (cat. No. M1210-2, 1:5,000 dilution, HuaAn Biotechnology, China), HPRT1 (cat. No.109021, 1:10,000 dilution, Abcam, UK). The Reh and resistant cells were treated with 6-MP (1 μg/ml) or 6-TG (1 μg/ml) for 48h and analyzed by SDS–PAGE with the following antibodies: γ-H2AX (S139) (cat. 9718; 1:1,000 dilution, Cell Signaling Technology (CST), USA), H2AX (cat. 7631; 1:1,000 dilution, CST, USA), phospho-CHK2 (T68) (cat. 1538-1; 1:1,000 dilution, CST, USA), CHK2 (cat. 3428-1; 1:50,000 dilution, Epitomics, USA), PARP (cat. 9542; 1:1,000 dilution, CST, USA), cleaved PARP (cat. 5625; 1:1,000 dilution, CST, USA) and GAPDH (cat. No. R1208-3, 1:5,000 dilution, HuaAn Biotechnology, China). Immunoblots were analyzed using the Odyssey system (LI-COR Biosciences, USA).

### Real-time quantitative RT-PCR assay

Total cellular RNA was isolated from cells using trizol extraction method. For RT-PCR, 1μg total RNA of the sample was used for cDNA synthesis by using PrimeScript^TM^ RT reagent kit with gDNA Eraser (Takara, cat. No. RR047A). The primer sequence of MDR1 was: sense 5’-CGACAGGAGATAGGCTGGTT-3’ and antisense 5’- GGTTAGCTTCCAACCACGTG-3’. The primer sequence of the internal control actin was: sense 5’-TGACGTGGACATCCGCAAAG-3’ and antisense 5’-CTGGAAGGTGGACAGCGAGG-3’. Real-time quantitative PCR was carried out for 40 cycles as follows: initial denaturation at 94°C for 5 min, template denaturation at 94°C for 30 sec, primer annealing at 60°C for 30 sec and primer extension/elongation at 72°C for 30 sec. The relative expression (RE) was calculated by the 2^-ΔΔCt^ method [18].

### Statistical analysis

Each result was repeated at least three times. The data were expressed as mean ± SD and analyzed by the Student's *t*-test, P<0.05 was considered to be statistically significant.

## SUPPLEMENTARY MATERIALS FIGURES AND TABLES






