# Supplementary material for: HPRT1 activity loss is associated with resistance to thiopurine in ALL
Source: Oncotarget. 2017 Dec 19;9(2):2268–78. doi: 10.18632/oncotarget.23405 (PMC5788638; doi:10.18632/oncotarget.23405)
Supplement: Supplementary file 1 [file oncotarget-09-2268-s001.pdf]

HPRT1 activity loss is associated with resistance to thiopurine in ALL

SUPPLEMENTARY MATERIALS

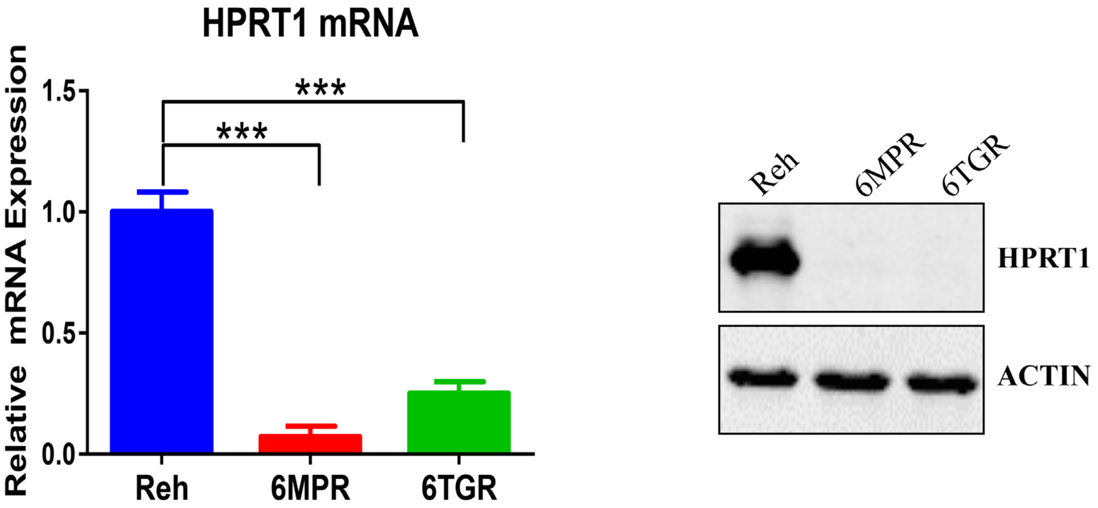

Supplementary Figure 1: The RNA and protein level of HPRT1 were measured by Real-time quantitative RT-PCR assay and western blot. Data are expressed as mean ± SD. ***P<0.001 compared with ctrl Reh group.

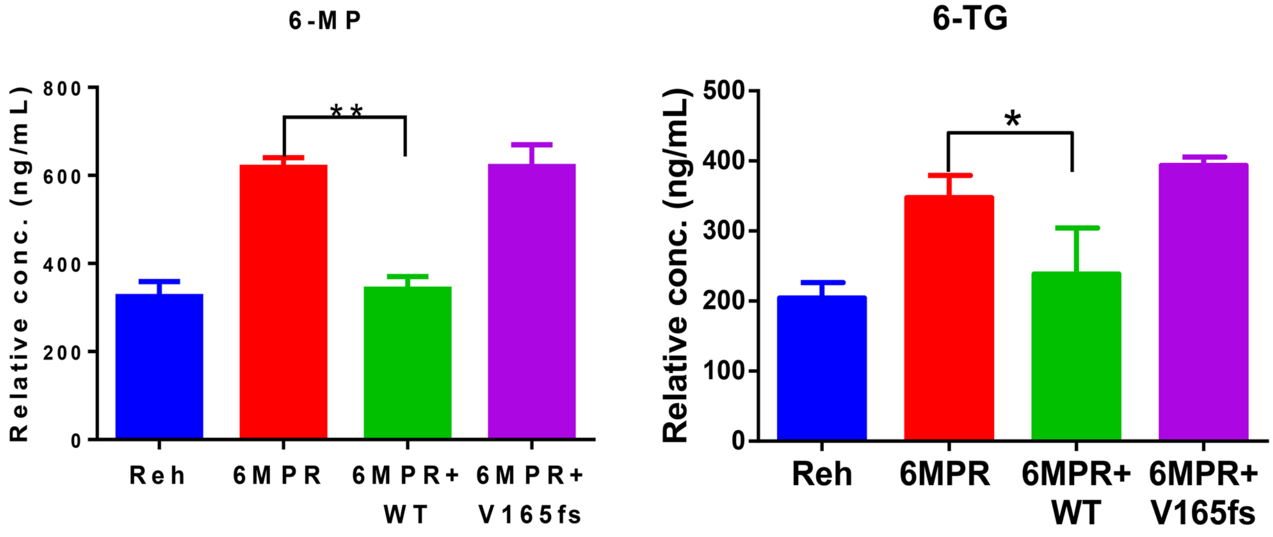

Supplementary Figure 2: The metabolism of 6-MP and 6-TG were measured by LC-MS in resistant cells and HPRT1 re-expression cells. Data are expressed as mean ± SD. *P<0.05, **P<0.01 compared with ctrl Reh group.

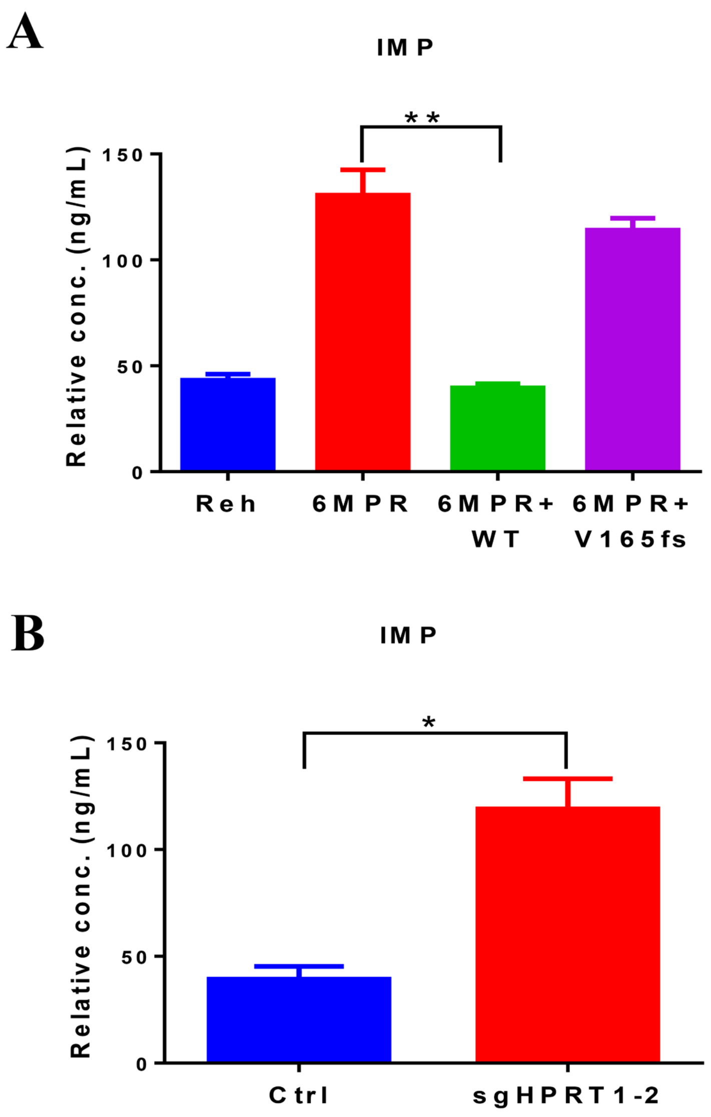

Supplementary Figure 3: (A) The metabolism of IMP were measured by LC-MS in resistant cells and HPRT1 re-expression cells. **(B)** The metabolism of IMP were measured by LC-MS in ctrl cells and HPRT1 Knockdown cells. Data are expressed as mean ± SD. *P<0.05, **P<0.01 compared with ctrl group.

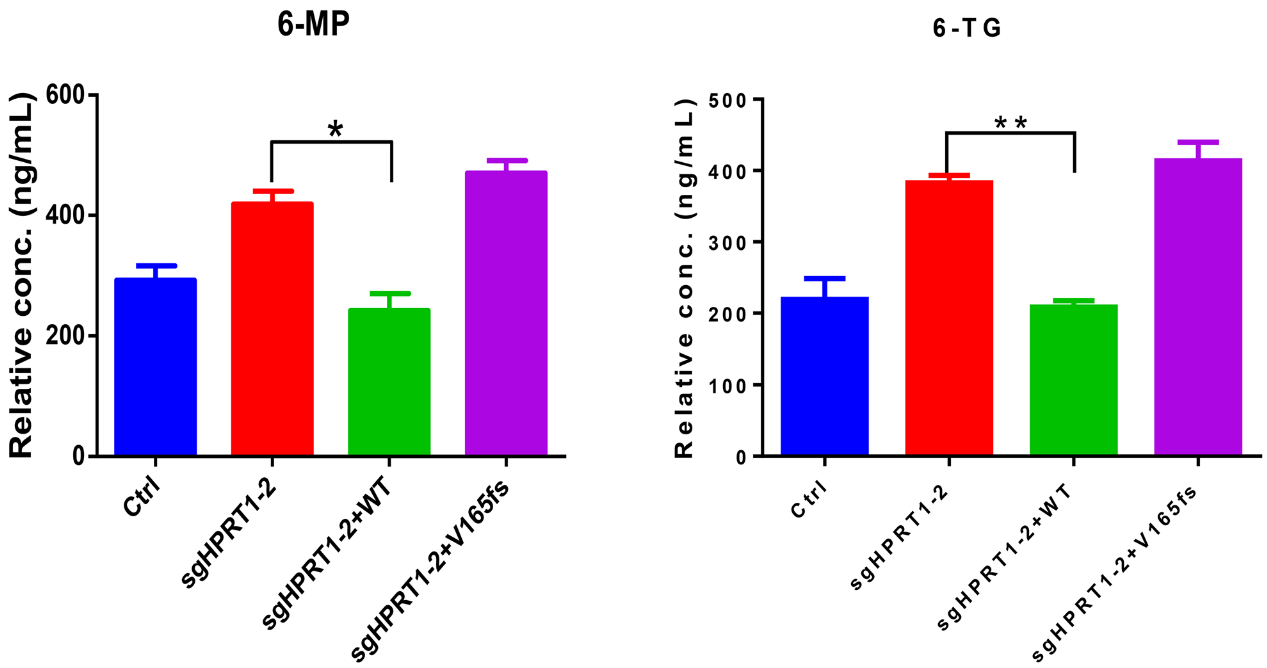

Supplementary Figure 4: The metabolism of 6-MP and 6-TG were measured by LC-MS in ctrl cells and HPRT1 Knockdown cells. Data are expressed as mean ± SD. $^{*}P<0.05$, $^{**}P<0.01$ compared with ctrl group.

Supplementary Table 1: The list of mutations in the Reh-6MPR cells.

See Supplementary File 1

Supplementary Table 2: The list of mutations in the Reh-6TGR cells.

See Supplementary File 2
