# Supplementary material for: HPRT1 activity loss is associated with resistance to thiopurine in ALL
Source: Oncotarget. 2017 Dec 19;9(2):2268–78. doi: 10.18632/oncotarget.23405 (PMC5788638; doi:10.18632/oncotarget.23405)
Supplement: Supplementary file 2 [file oncotarget-09-2268-s002.docx]

**Supplementary Table 1: The list of mutations in the Reh-6MPR cells**

| **Gene Name** | **Position (in hg19)** | **Mutated type** | **Ref Allele** | **Var Allele** | **ref reads** | **var reads** | **total reads** | **Var Freq** | **Reh total reads** | **Predicted amino acid change** |
| --- | --- | --- | --- | --- | --- | --- | --- | --- | --- | --- |
| ABL1 | chr9:133760232 | frameshift deletion | C | - | 100 | 105 | 205 | 51.22% | 273 | p.T852fs |
| ADCY2 | chr5:7690847 | nonsynonymous SNV | A | C | 37 | 39 | 76 | 51.32% | 91 | p.E255A |
| ANAPC5 | chr12:121766213 | nonsynonymous SNV | C | T | 26 | 36 | 62 | 58.06% | 65 | p.D292N |
| ARID1A | chr1:27099406 | nonsynonymous SNV | A | G | 28 | 34 | 62 | 54.84% | 40 | p.M1215V |
| ARID2 | chr12:46245157 | nonsynonymous SNV | T | A | 45 | 34 | 79 | 43.04% | 64 | p.I1084N |
| ARID4B | chr1:235377302 | frameshift insertion | - | G | 43 | 11 | 54 | 20.37% | 25 | p.E541fs |
| ARID5A | chr2:97217671 | nonsynonymous SNV | C | T | 20 | 30 | 50 | 60.00% | 99 | p.A469V |
| ATP2A1 | chr16:28913350 | nonsynonymous SNV | A | G | 87 | 57 | 144 | 39.58% | 175 | p.N756S |
| ATP2A3 | chr17:3850789 | nonsynonymous SNV | C | T | 358 | 130 | 488 | 26.64% | 521 | p.A331T |
| AVP | chr20:3063377 | nonsynonymous SNV | C | T | 28 | 16 | 44 | 36.36% | 29 | p.A132T |
| BCAN | chr1:156616599 | nonsynonymous SNV | G | A | 35 | 42 | 77 | 54.55% | 89 | p.R33H |
| BRPF3 | chr6:36189970 | nonsynonymous SNV | C | T | 79 | 93 | 172 | 54.07% | 213 | p.R1037C |
| C10orf137 | chr10:127436489 | nonsynonymous SNV | T | G | 24 | 33 | 57 | 57.89% | 56 | p.S977A |
| C19orf35 | chr19:2279100 | nonsynonymous SNV | G | A | 35 | 24 | 59 | 40.68% | 85 | p.A32V |
| C20orf195 | chr20:62187797 | nonsynonymous SNV | A | G | 142 | 136 | 278 | 48.92% | 321 | p.I261V |
| CACNA1F | chrX:49069198 | nonsynonymous SNV | G | A | 21 | 35 | 56 | 62.50% | 40 | p.R1291W |
| CACNA1I | chr22:40058177 | nonsynonymous SNV | G | A | 24 | 24 | 48 | 50.00% | 75 | p.A1002T |
| CACTIN | chr19:3613119 | nonsynonymous SNV | C | T | 49 | 21 | 70 | 30.00% | 108 | p.V575M |
| CAGE1 | chr6:7374042 | frameshift deletion | AC | - | 24 | 7 | 31 | 22.58% | 27 | p.201_201del |
| CASKIN1 | chr16:2230692 | nonsynonymous SNV | G | A | 69 | 81 | 150 | 54.00% | 170 | p.P893S |
| CEACAM3 | chr19:42301581 | nonsynonymous SNV | C | A | 114 | 41 | 155 | 26.45% | 152 | p.P42Q |
| CEBPZ | chr2:37449570 | nonframeshift deletion | GTT | - | 34 | 8 | 42 | 19.05% | 24 | p.672_673del |
| CIT | chr12:120128143 | nonsynonymous SNV | C | T | 79 | 29 | 108 | 26.85% | 97 | p.R1958H |
| CNGA3 | chr2:98996748 | nonsynonymous SNV | A | G | 98 | 19 | 117 | 16.24% | 116 | p.E109G |
| CPNE7 | chr16:89642340 | frameshift deletion | C | - | 24 | 13 | 37 | 35.14% | 39 | p.T12fs |
| CSMD1 | chr8:2832078 | nonsynonymous SNV | C | T | 100 | 66 | 166 | 39.76% | 187 | p.A2879T |
| CST1 | chr20:23729754 | stopgain SNV | C | - | 29 | 16 | 45 | 35.56% | 58 | p.V81X |
| CTU2 | chr16:88780888 | nonsynonymous SNV | G | A | 282 | 52 | 334 | 15.57% | 407 | p.A399T |
| DISP2 | chr15:40661570 | nonsynonymous SNV | G | A | 78 | 24 | 102 | 23.53% | 110 | p.R1086H |
| DNAH6 | chr2:84752796 | frameshift deletion | A | - | 14 | 5 | 19 | 26.32% | 18 | p.A111fs |
| DNAH9 | chr17:11502171 | nonsynonymous SNV | C | T | 44 | 52 | 96 | 54.17% | 105 | p.A119V |
| DRAP1 | chr11:65688801 | frameshift insertion | - | G | 21 | 5 | 26 | 19.23% | 28 | p.S171fs |
| EBF2 | chr8:25898490 | nonsynonymous SNV | C | T | 85 | 39 | 124 | 31.45% | 143 | p.G106D |
| EML4 | chr2:42488302 | frameshift deletion | A | - | 14 | 1 | 15 | 6.67% | 12 | p.E127fs |
| ESPL1 | chr12:53679877 | nonsynonymous SNV | A | G | 48 | 37 | 85 | 43.53% | 132 | p.I1119M |
| FASN | chr17:80039512 | nonsynonymous SNV | T | C | 204 | 55 | 259 | 21.24% | 222 | p.Q2124R |
| GP5 | chr3:194118986 | nonsynonymous SNV | G | A | 60 | 30 | 90 | 33.33% | 111 | p.A9V |
| GPR123 | chr10:134942785 | nonsynonymous SNV | G | A | 94 | 70 | 164 | 42.68% | 215 | p.E485K |
| GPR25 | chr1:200842473 | nonsynonymous SNV | G | A | 48 | 50 | 98 | 51.02% | 77 | p.G103D |
| HEXIM2 | chr17:43246989 | nonsynonymous SNV | C | T | 241 | 62 | 303 | 20.46% | 369 | p.S225L |
| HHIPL2 | chr1:222713535 | nonsynonymous SNV | G | A | 246 | 50 | 296 | 16.89% | 321 | p.R423C |
| HPRT1 | chrX:133632429 | frameshift insertion | - | A | 1 | 15 | 16 | 93.75% | 14 | p.V165fs |
| HRH3 | chr20:60794909 | nonsynonymous SNV | G | A | 33 | 47 | 80 | 58.75% | 105 | p.L40F |
| JMJD1C | chr10:64950784 | nonsynonymous SNV | G | T | 58 | 22 | 80 | 27.50% | 44 | p.P1817H |
| KCNMA1 | chr10:78771791 | frameshift deletion | A | - | 17 | 11 | 28 | 39.29% | 29 | p.Y676fs |
| KLF1 | chr19:12996830 | nonsynonymous SNV | T | A | 75 | 81 | 156 | 51.92% | 188 | p.T72S |
| KRT77 | chr12:53096990 | frameshift insertion | - | C | 27 | 11 | 38 | 28.95% | 36 | p.R77fs |
| LCN8 | chr9:139649905 | nonsynonymous SNV | A | T | 162 | 131 | 293 | 44.71% | 337 | p.L97Q |
| LHX1 | chr17:35295508 | nonsynonymous SNV | C | A | 100 | 81 | 181 | 44.75% | 204 | p.A5D |
| LRP1 | chr12:57569833 | nonsynonymous SNV | A | G | 152 | 157 | 309 | 50.81% | 409 | p.Y1312C |
| MFSD10 | chr4:2934912 | nonsynonymous SNV | A | G | 192 | 50 | 242 | 20.66% | 297 | p.V98A |
| MLL | chr11:118390479 | nonsynonymous SNV | G | A | 40 | 8 | 48 | 16.67% | 54 | p.G3765S |
| MNS1 | chr15:56736723 | frameshift deletion | T | - | 52 | 18 | 70 | 25.71% | 32 | p.K202fs |
| MTSS1L | chr16:70697995 | nonsynonymous SNV | G | A | 155 | 144 | 299 | 48.16% | 367 | p.A610V |
| MUC16 | chr19:9073953 | nonsynonymous SNV | T | C | 22 | 27 | 49 | 55.10% | 50 | p.N4498S |
| MYO16 | chr13:109792935 | nonsynonymous SNV | T | C | 115 | 22 | 137 | 16.06% | 149 | p.C1459R |
| NARF | chr17:80441648 | nonsynonymous SNV | G | A | 42 | 21 | 63 | 33.33% | 63 | p.D228N |
| PANX1 | chr11:93913373 | nonsynonymous SNV | T | C | 24 | 33 | 57 | 57.89% | 49 | p.M384T |
| PCYOX1 | chr2:70502206 | stopgain SNV | C | T | 27 | 25 | 52 | 48.08% | 69 | p.Q204X |
| PHLDA1 | chr12:76425342 | nonsynonymous SNV | T | A | 47 | 50 | 97 | 51.55% | 117 | p.R60S |
| PLEKHG5 | chr1:6556564 | nonsynonymous SNV | G | A | 59 | 39 | 98 | 39.80% | 120 | p.R24W |
| POLG | chr15:89876859 | nonframeshift deletion | GCC | - | 172 | 127 | 299 | 42.47% | 344 | p.42_43del |
| POPDC3 | chr6:105606527 | frameshift deletion | A | - | 4 | 6 | 10 | 60.00% | 6 | p.S232fs |
| RALGDS | chr9:135977885 | nonsynonymous SNV | C | A | 203 | 167 | 370 | 45.14% | 372 | p.A608S |
| RAPGEF3 | chr12:48131436 | nonsynonymous SNV | C | T | 64 | 51 | 115 | 44.35% | 152 | p.A854T |
| RBP3 | chr10:48390369 | nonsynonymous SNV | C | T | 87 | 78 | 165 | 47.27% | 229 | p.R170Q |
| RET | chr10:43606691 | nonsynonymous SNV | A | G | 31 | 46 | 77 | 59.74% | 86 | p.S434G |
| RPAP3 | chr12:48096583 | nonsynonymous SNV | A | G | 23 | 23 | 46 | 50.00% | 37 | p.V14A |
| SALL3 | chr18:76755310 | nonsynonymous SNV | C | T | 42 | 16 | 58 | 27.59% | 85 | p.R1107C |
| SAMD4A | chr14:55241701 | nonsynonymous SNV | G | T | 92 | 34 | 126 | 26.98% | 165 | p.G180C |
| SBNO1 | chr12:123805299 | nonsynonymous SNV | C | T | 18 | 18 | 36 | 50.00% | 29 | p.R813Q |
| SCN2A | chr2:166152433 | nonsynonymous SNV | G | T | 31 | 32 | 63 | 50.79% | 91 | p.A34S |
| SEC16A | chr9:139370710 | nonsynonymous SNV | C | T | 35 | 36 | 71 | 50.70% | 75 | p.G453D |
| SERPINA6 | chr14:94780651 | nonsynonymous SNV | A | G | 163 | 46 | 209 | 22.01% | 204 | p.L112P |
| SH3RF2 | chr5:145439492 | nonsynonymous SNV | G | A | 80 | 60 | 140 | 42.86% | 188 | p.R540H |
| SIPA1L1 | chr14:72202008 | frameshift deletion | T | - | 34 | 9 | 43 | 20.93% | 38 | p.F1696fs |
| SLC22A4 | chr5:131630574 | nonsynonymous SNV | A | G | 77 | 78 | 155 | 50.32% | 221 | p.I89V |
| SLITRK6 | chr13:86369013 | nonsynonymous SNV | A | T | 20 | 27 | 47 | 57.45% | 31 | p.I544N |
| SLX4 | chr16:3633171 | nonsynonymous SNV | C | T | 144 | 113 | 257 | 43.97% | 305 | p.A1694T |
| SMARCD3 | chr7:150939589 | nonsynonymous SNV | A | G | 45 | 54 | 99 | 54.55% | 129 | p.V186A |
| SOGA3 | chr6:127836936 | nonsynonymous SNV | G | A | 230 | 48 | 278 | 17.27% | 352 | p.A275V |
| SPG11 | chr15:44907750 | frameshift deletion | A | - | 12 | 9 | 21 | 42.86% | 35 | p.L950fs |
| SPTB | chr14:65260096 | nonsynonymous SNV | G | A | 164 | 176 | 340 | 51.76% | 454 | p.A762V |
| STAM | chr10:17750839 | nonsynonymous SNV | T | A | 58 | 41 | 99 | 41.41% | 107 | p.L425H |
| SV2B | chr15:91832791 | frameshift insertion | - | T | 29 | 22 | 51 | 43.14% | 60 | p.L432fs |
| TNNT1 | chr19:55652558 | nonsynonymous SNV | C | T | 52 | 24 | 76 | 31.58% | 91 | p.R91H |
| TOX2 | chr20:42635399 | nonsynonymous SNV | G | T | 133 | 101 | 234 | 43.16% | 238 | p.Q84H |
| TPI1 | chr12:6978870 | nonsynonymous SNV | G | T | 50 | 44 | 94 | 46.81% | 97 | p.S159I |
| TTN | chr2:179393748 | frameshift deletion | T | - | 21 | 7 | 28 | 25.00% | 29 | p.K26512fs |
| TYMS | chr18:670748 | nonsynonymous SNV | A | G | 72 | 99 | 171 | 57.89% | 188 | p.N205D |
| U2AF1L4 | chr19:36233701 | nonframeshift deletion | TGA | - | 30 | 11 | 41 | 26.83% | 37 | p.155_155del |
| USP40 | chr2:234434195 | frameshift insertion | - | A | 20 | 11 | 31 | 35.48% | 32 | p.W591fs |
| USP5 | chr12:6973089 | nonsynonymous SNV | G | A | 101 | 85 | 186 | 45.70% | 180 | p.G684R |
| WDR96 | chr10:105971767 | nonsynonymous SNV | G | A | 57 | 20 | 77 | 25.97% | 46 | p.R245W |
| ZFPM1 | chr16:88601338 | nonsynonymous SNV | C | T | 32 | 19 | 51 | 37.25% | 66 | p.A991V |
| ZNF14 | chr19:19823077 | nonsynonymous SNV | T | C | 26 | 26 | 52 | 50.00% | 47 | p.K338R |
| ZNF585B | chr19:37677631 | nonsynonymous SNV | A | G | 70 | 58 | 128 | 45.31% | 124 | p.Y270H |
| ZNF681 | chr19:23926523 | nonsynonymous SNV | T | C | 46 | 9 | 55 | 16.36% | 53 | p.K610R |
| ZSWIM8 | chr10:75548553 | nonsynonymous SNV | A | G | 43 | 25 | 68 | 36.76% | 50 | p.S112G |
