# Supplementary material for: HPRT1 activity loss is associated with resistance to thiopurine in ALL
Source: Oncotarget. 2017 Dec 19;9(2):2268–78. doi: 10.18632/oncotarget.23405 (PMC5788638; doi:10.18632/oncotarget.23405)
Supplement: Supplementary file 3 [file oncotarget-09-2268-s003.docx]

**Supplementary Table 2: The list of mutations in the Reh-6TGR cells**

| **Gene Name** | **Position (in hg19)** | **Mutated type** | **Ref Allele** | **Var Allele** | **ref reads** | **var reads** | **total reads** | **Var Freq** | **Reh total reads** | **Predicted amino acid change** |
| --- | --- | --- | --- | --- | --- | --- | --- | --- | --- | --- |
| ADCY2 | chr5:7690847 | nonsynonymous SNV | A | C | 42 | 26 | 68 | 38.24% | 91 | p.E255A |
| AKAP9 | chr3:37366569 | frameshift deletion | G | - | 16 | 8 | 24 | 33.33% | 10 | p.E727fs |
| ARID2 | chr12:46245157 | nonsynonymous SNV | T | A | 36 | 30 | 66 | 45.45% | 64 | p.I1084N |
| ARID4B | chr20:13763500 | frameshift insertion | - | G | 25 | 6 | 31 | 19.35% | 20 | p.E541fs |
| ARID5A | chr2:97217671 | nonsynonymous SNV | C | T | 36 | 25 | 61 | 40.98% | 99 | p.A469V |
| ATP2A1 | chr16:28913350 | nonsynonymous SNV | A | G | 69 | 38 | 107 | 35.51% | 175 | p.N756S |
| BCAN | chr1:156616599 | nonsynonymous SNV | G | A | 39 | 23 | 62 | 37.10% | 89 | p.R33H |
| BRPF3 | chr6:36189970 | nonsynonymous SNV | C | T | 87 | 45 | 132 | 34.09% | 213 | p.R1037C |
| C10orf137 | chr10:127436489 | nonsynonymous SNV | T | G | 27 | 25 | 52 | 48.08% | 56 | p.S977A |
| C20orf195 | chr20:62187797 | nonsynonymous SNV | A | G | 133 | 105 | 238 | 44.12% | 321 | p.I261V |
| CASKIN1 | chr16:2230692 | nonsynonymous SNV | G | A | 61 | 40 | 101 | 39.60% | 170 | p.P893S |
| CLN6 | chr15:68504189 | nonsynonymous SNV | A | G | 45 | 14 | 59 | 23.73% | 89 | p.S104P |
| CPNE7 | chr2:234434195 | frameshift deletion | C | - | 10 | 4 | 14 | 28.57% | 32 | p.T12fs |
| CRYZL1 | chr10:78771791 | frameshift deletion | AA | - | 11 | 10 | 21 | 47.62% | 29 | p.347_347del |
| CSMD1 | chr8:2832078 | nonsynonymous SNV | C | T | 81 | 34 | 115 | 29.57% | 187 | p.A2879T |
| CYLC2 | chr2:42488302 | frameshift deletion | G | - | 17 | 9 | 26 | 34.62% | 12 | p.K337fs |
| DHX8 | chr17:41584941 | nonsynonymous SNV | T | C | 44 | 10 | 54 | 18.52% | 69 | p.M665T |
| DNAH9 | chr17:11502171 | nonsynonymous SNV | C | T | 29 | 29 | 58 | 50.00% | 105 | p.A119V |
| DNAJC11 | chr1:6697540 | stopgain SNV | G | A | 60 | 17 | 77 | 22.08% | 127 | p.R450X |
| EIF5B | chr2:99977966 | frameshift insertion | - | GGGC | 55 | 6 | 61 | 9.84% | 47 | p.K201fs |
| EML4 | chr6:108214705 | frameshift deletion | A | - | 15 | 9 | 24 | 37.50% | 19 | p.E127fs |
| ERCC4 | chr1:93704996 | frameshift deletion | GT | - | 14 | 5 | 19 | 26.32% | 18 | p.449_449del |
| ESPL1 | chr12:53679877 | nonsynonymous SNV | A | G | 40 | 20 | 60 | 33.33% | 132 | p.I1119M |
| FAM114A1 | chr2:176957790 | frameshift insertion | - | G | 21 | 5 | 26 | 19.23% | 26 | p.K431fs |
| FHDC1 | chr6:7374042 | frameshift insertion | - | T | 22 | 6 | 28 | 21.43% | 27 | p.S57fs |
| GOLGA4 | chr17:16455906 | frameshift deletion | C | - | 35 | 14 | 49 | 28.57% | 33 | p.I1086fs |
| GPR123 | chr10:134942785 | nonsynonymous SNV | G | A | 94 | 34 | 128 | 26.56% | 215 | p.E485K |
| GPR25 | chr1:200842473 | nonsynonymous SNV | G | A | 41 | 34 | 75 | 45.33% | 77 | p.G103D |
| HPRT1 | chrX:133632429 | frameshift insertion | - | A | 3 | 8 | 11 | 72.73% | 14 | p.V165fs |
| HRH3 | chr20:60794909 | nonsynonymous SNV | G | A | 33 | 25 | 58 | 43.10% | 105 | p.L40F |
| KLF1 | chr19:12996830 | nonsynonymous SNV | T | A | 56 | 55 | 111 | 49.55% | 188 | p.T72S |
| KRT77 | chr6:26409825 | frameshift deletion | AAAC | - | 46 | 7 | 53 | 13.21% | 66 | p.111_112del |
| LHX1 | chr17:35295508 | nonsynonymous SNV | C | A | 77 | 48 | 125 | 38.40% | 204 | p.A5D |
| LRP1 | chr12:57569833 | nonsynonymous SNV | A | G | 158 | 98 | 256 | 38.28% | 409 | p.Y1312C |
| MAP1B | chr3:47452185 | nonframeshift deletion | TTA | - | 31 | 6 | 37 | 16.22% | 34 | p.843_843del |
| MEGF9 | chr1:41481815 | nonframeshift deletion | CGGCGG | - | 48 | 40 | 88 | 45.45% | 127 | p.30_32del |
| MKI67 | chr10:129902758 | nonframeshift deletion | TGA | - | 190 | 30 | 220 | 13.64% | 216 | p.2088_2089del |
| MKI67 | chr10:129913846 | nonsynonymous SNV | T | C | 33 | 23 | 56 | 41.07% | 66 | p.S276G |
| MKI67 | chr13:103384036 | frameshift deletion | T | - | 16 | 5 | 21 | 23.81% | 21 | p.L1574fs |
| MTSS1L | chr16:70697995 | nonsynonymous SNV | G | A | 159 | 92 | 251 | 36.65% | 367 | p.A610V |
| MUC4 | chr1:93705004 | frameshift deletion | A | - | 14 | 5 | 19 | 26.32% | 17 | p.S104fs |
| NLRP3 | chr1:247611776 | nonsynonymous SNV | G | T | 46 | 15 | 61 | 24.59% | 102 | p.E913D |
| OR6C1 | chr15:91832791 | frameshift insertion | - | A | 29 | 16 | 45 | 35.56% | 60 | p.T8fs |
| PANX1 | chr11:93913373 | nonsynonymous SNV | T | C | 34 | 18 | 52 | 34.62% | 49 | p.M384T |
| PHLDA1 | chr12:76425342 | nonsynonymous SNV | T | A | 54 | 35 | 89 | 39.33% | 117 | p.R60S |
| PLEKHG5 | chr1:6556564 | nonsynonymous SNV | G | A | 52 | 28 | 80 | 35.00% | 120 | p.R24W |
| POLG | chr15:89876856 | nonframeshift deletion | GCTGCC | - | 224 | 9 | 233 | 3.86% | 347 | p.42_44del |
| POLQ | chr3:121264671 | nonsynonymous SNV | A | T | 79 | 43 | 122 | 35.25% | 201 | p.D18E |
| PRG4 | chr1:186277638 | nonframeshift insertion | - | ATG | 79 | 12 | 91 | 13.19% | 56 | p.E795del |
| RAPGEF3 | chr12:48131436 | nonsynonymous SNV | C | T | 48 | 26 | 74 | 35.14% | 152 | p.A854T |
| RBP3 | chr10:48390369 | nonsynonymous SNV | C | T | 70 | 50 | 120 | 41.67% | 229 | p.R170Q |
| RET | chr10:43606691 | nonsynonymous SNV | A | G | 41 | 25 | 66 | 37.88% | 86 | p.S434G |
| S100A1 | chr20:31384651 | frameshift deletion | A | - | 64 | 16 | 80 | 20.00% | 117 | p.D53fs |
| SARM1 | chr17:26708728 | nonsynonymous SNV | A | G | 33 | 18 | 51 | 35.29% | 62 | p.D325G |
| SCN2A | chr2:166152433 | nonsynonymous SNV | G | T | 44 | 30 | 74 | 40.54% | 91 | p.A34S |
| SEC16A | chr9:139370710 | nonsynonymous SNV | C | T | 25 | 32 | 57 | 56.14% | 75 | p.G453D |
| SGOL2 | chr7:19738020 | nonframeshift deletion | AAG | - | 17 | 9 | 26 | 34.62% | 23 | p.710_710del |
| SH3RF2 | chr5:145439492 | nonsynonymous SNV | G | A | 91 | 32 | 123 | 26.02% | 188 | p.R540H |
| SLC22A10 | chr11:64375204 | frameshift insertion | - | A | 149 | 90 | 239 | 37.66% | 352 | p.N536fs |
| SLC22A4 | chr5:131630574 | nonsynonymous SNV | A | G | 87 | 48 | 135 | 35.56% | 221 | p.I89V |
| SLX4 | chr16:3633171 | nonsynonymous SNV | C | T | 136 | 71 | 207 | 34.30% | 305 | p.A1694T |
| SMARCD3 | chr7:150939589 | nonsynonymous SNV | A | G | 53 | 36 | 89 | 40.45% | 129 | p.V186A |
| SP9 | chr2:175201316 | nonsynonymous SNV | C | T | 32 | 23 | 55 | 41.82% | 67 | p.A168V |
| STAM | chr10:17750839 | nonsynonymous SNV | T | A | 50 | 30 | 80 | 37.50% | 107 | p.L425H |
| SV2B | chr2:201437197 | frameshift insertion | - | T | 20 | 9 | 29 | 31.03% | 29 | p.L583fs |
| TNNT1 | chr19:55652558 | nonsynonymous SNV | C | T | 48 | 20 | 68 | 29.41% | 91 | p.R91H |
| TPI1 | chr12:6978870 | nonsynonymous SNV | G | T | 39 | 28 | 67 | 41.79% | 97 | p.S159I |
| TRIM21 | chr11:4409593 | nonframeshift deletion | GGC | - | 28 | 5 | 33 | 15.15% | 49 | p.224_224del |
| TYMS | chr18:670748 | nonsynonymous SNV | A | G | 84 | 46 | 130 | 35.38% | 188 | p.N205D |
| USP5 | chr12:6973089 | nonsynonymous SNV | G | A | 93 | 58 | 151 | 38.41% | 180 | p.G684R |
| VWDE | chr12:53096990 | stopgain SNV | - | A | 20 | 9 | 29 | 31.03% | 36 | p.K1273_E1274del |
| ZKSCAN1 | chr7:99630937 | frameshift insertion | - | C | 32 | 6 | 38 | 15.79% | 31 | p.N270fs |
| ZNF493 | chr19:21606179 | nonframeshift insertion | - | GTG | 34 | 5 | 39 | 12.82% | 50 | p.Y112del |
| ZNF585B | chr19:37677631 | nonsynonymous SNV | A | G | 64 | 41 | 105 | 39.05% | 124 | p.Y270H |
| ZNF701 | chr19:53086139 | nonsynonymous SNV | G | A | 81 | 15 | 96 | 15.63% | 103 | p.G276D |
